# Association Analysis Between the Functional Single Nucleotide Variants in miR-146a, miR-196a-2, miR-499a, and miR-612 With Acute Lymphoblastic Leukemia

**DOI:** 10.3389/fonc.2021.762063

**Published:** 2021-11-05

**Authors:** Silvia Jiménez-Morales, Juan Carlos Núñez-Enríquez, Jazmín Cruz-Islas, Vilma Carolina Bekker-Méndez, Elva Jiménez-Hernández, Aurora Medina-Sanson, Irma Olarte-Carrillo, Adolfo Martínez-Tovar, Janet Flores-Lujano, Julian Ramírez-Bello, María Luisa Pérez-Saldívar, Jorge Alfonso Martín-Trejo, Héctor Pérez-Lorenzana, Raquel Amador-Sánchez, Felix Gustavo Mora-Ríos, José Gabriel Peñaloza-González, David Aldebarán Duarte-Rodríguez, José Refugio Torres-Nava, Juan Eduardo Flores-Bautista, Rosa Martha Espinosa-Elizondo, Pedro Francisco Román-Zepeda, Luz Victoria Flores-Villegas, Edna Liliana Tamez-Gómez, Víctor Hugo López-García, José Ramón Lara-Ramos, Juana Esther González-Ulivarri, Sofía Irene Martínez-Silva, Gilberto Espinoza-Anrubio, Carolina Almeida-Hernández, Rosario Ramírez-Colorado, Luis Hernández-Mora, Luis Ramiro García-López, Gabriela Adriana Cruz-Ojeda, Arturo Emilio Godoy-Esquivel, Iris Contreras-Hernández, Abraham Medina-Hernández, María Guadalupe López-Caballero, Norma Angélica Hernández-Pineda, Jorge Granados-Kraulles, María Adriana Rodríguez-Vázquez, Delfino Torres-Valle, Carlos Cortés-Reyes, Francisco Medrano-López, Jessica Arleet Pérez-Gómez, Annel Martínez-Ríos, Antonio Aguilar-De-los-Santos, Berenice Serafin-Díaz, María de Lourdes Gutiérrez-Rivera, Laura Elizabeth Merino-Pasaye, Gilberto Vargas-Alarcón, Minerva Mata-Rocha, Omar Alejandro Sepúlveda-Robles, Haydeé Rosas-Vargas, Alfredo Hidalgo-Miranda, Juan Manuel Mejía-Aranguré

**Affiliations:** ^1^ Laboratorio de Genómica del Cáncer, Instituto Nacional de Medicina Genómica, Mexico City, Mexico; ^2^ Unidad de Investigación Médica en Epidemiología Clínica, Unidad Medica de Alta Especialidad (UMAE) Hospital de Pediatría “Dr. Silvestre Frenk Freund”, Centro Médico Nacional “Siglo XXI”, Instituto Mexicano del Seguro Social (IMSS), Mexico City, Mexico; ^3^ Unidad de Investigación Médica en Inmunología e Infectología, Hospital de Infectología “Dr. Daniel Méndez Hernández”, “La Raza”, IMSS, Mexico City, Mexico; ^4^ Servicio de Hematología Pediátrica, Hospital General “Gaudencio González Garza”, Centro Médico Nacional “La Raza”, IMSS, Mexico City, Mexico; ^5^ Departamento de Hemato-Oncología, Hospital Infantil de México Federico Gómez, Mexico City, Mexico; ^6^ Servicio de Hematología, Departamento de Investigación, Hospital General de México Dr. Eduardo Liceaga, Mexico City, Mexico; ^7^ Departamento de Endocrinología, Instituto Nacional de Cardiología, Ignacio Chávez, México City, Mexico; ^8^ Servicio de Hematología Pediátrica Unidad Medica de Alta Especialidad (UMAE) Hospital de Pediatría “Dr. Silvestre Frenk Freund”, Centro Médico Nacional “Siglo XXI”, IMSS, Mexico City, Mexico; ^9^ Servicio de Cirugía Pediátrica, Hospital General “Gaudencio González Garza”, Centro Médico Nacional (CMN) “La Raza”, IMSS, Mexico City, Mexico; ^10^ Servicio de Hematología Pediátrica, Hospital General Regional “Carlos McGregor Sánchez Navarro”, IMSS, Mexico City, Mexico; ^11^ Cirugía Pediátrica, Hospital Regional “General Ignacio Zaragoza”, Instituto de Seguridad y Servicios Sociales de los Trabajadores del Estado (ISSSTE), Mexico City, Mexico; ^12^ Servicio de Onco-Pediatria, Hospital Juárez de México, Mexico City, Mexico; ^13^ Servicio de Oncología, Hospital Pediátrico de Moctezuma, Secretaría de Salud de la Ciudad de México (SSCDMX), Mexico City, Mexico; ^14^ Servicio de Pediatría, Hospital General de Tláhuac, Mexico City, Mexico; ^15^ Servicio de Hematología Pediátrica, Hospital General de México, Mexico City, Mexico; ^16^ Coordinación Clínica y Servicio de Cirugía Pediátrica, Hospital General Regional (HGR) No. 1 “Dr. Carlos Mac Gregor Sánchez Navarro”, IMSS, Mexico City, Mexico; ^17^ Servicio de Hematología Pediátrica, Centro Médico Nacional “20 de Noviembre”, ISSSTE, Mexico City, Mexico; ^18^ Servicio de Hemato-Oncología Hospital Infantil de Tamaulipas, Cd. Victoria, Mexico; ^19^ Jefatura de Enseñanza, Hospital Pediátrico de Iztacalco, SSCDMX, Mexico City, Mexico; ^20^ Jefatura de Enseñanza, Hospital Pediátrico de Iztapalapa, SSCDMX, Mexico City, Mexico; ^21^ Servicio de Pediatría, Hospital General Zona (HGZ) No. 8 “Dr. Gilberto Flores Izquierdo” IMSS, Mexico City, Mexico; ^22^ Jefatura de Enseñanza, Hospital General de Ecatepec “Las Américas”, Instituto de Salud del Estado de México (ISEM), Mexico City, Mexico; ^23^ Jefatura de Enseñanza, Hospital Pediátrico La Villa, SSCDMX, Mexico City, Mexico; ^24^ Jefatura de Enseñanza, Hospital Pediátrico San Juan de Aragón, Secretaría de Salud (SS), Mexico City, Mexico; ^25^ Servicio de Pediatría, Hospital Pediátrico de Tacubaya, SSCDMX, Mexico City, Mexico; ^26^ Coordinación Clínica de Educación e Investigación en Salud, HGZ No. 47, IMSS, Mexico City, Mexico; ^27^ Servicio de Cirugía Pediátrica, Hospital Pediátrico de Moctezuma, SSCDMX, Mexico City, Mexico; ^28^ Coordinación de Investigación en Salud, IMSS, Mexico City, Mexico; ^29^ Pediatría, Hospital Materno-Pediátrico de Xochimilco, SSCDMX, Mexico City, Mexico; ^30^ Jefatura de Enseñanza, Hospital Pediátrico de Coyoacán, SSCDMX, Mexico City, Mexico; ^31^ Coordinación Clínica y Pediatría del Hospital General de Zona 76, IMSS, Mexico City, Mexico; ^32^ Coordinación Clínica y Pediatría del Hospital General de Zona 68, IMSS, Mexico City, Mexico; ^33^ Coordinación Clínica y Pediatría del Hospital General de Zona 71, IMSS, Mexico City, Mexico; ^34^ Pediatría, Hospital General Dr. Darío Fernández Fierro, ISSSTE, Mexico City, Mexico; ^35^ Coordinación Clínica y Servicio de Pediatría, HGR No. 72 “Dr. Vicente Santos Guajardo”, IMSS, Mexico City, Mexico; ^36^ Cirugía Pediátrica del Hospital Regional “General Ignacio Zaragoza”, ISSSTE, Mexico City, Mexico; ^37^ Coordinación Clínica y Pediatría del Hospital General de Zona 98, IMSS, Mexico City, Mexico; ^38^ Coordinación Clínica y Pediatría del Hospital General de Zona 57, IMSS, Mexico City, Mexico; ^39^ Servicio de Oncología Pediátrica Unidad Medica de Alta Especialidad (UMAE) Hospital de Pediatría “Dr. Silvestre Frenk Freund”, IMSS, Mexico City, Mexico; ^40^ Departamento of Biología Molecular, Instituto Nacional de Cardiología Ignacio Chávez, Mexico City, Mexico; ^41^ Unidad de Investigación Médica en Genética Humana, Unidad Medica de Alta Especialidad (UMAE) Hospital de Pediatría “Dr. Silvestre Frenk Freund”, Centro Médico Nacional “Siglo XXI”, IMSS, Mexico City, Mexico; ^42^ Facultad de Medicina, Universidad Nacional Autónoma de México, Mexico City, Mexico

**Keywords:** acute lymphoblastic leukemia, *mir-146a*, *mir-196a-2*, *miR-499a*, *miR-612*, association study, Mexican population, single nucleotide polymorphism

## Abstract

**Background:**

Acute lymphoblastic leukemia (ALL) is characterized by an abnormal proliferation of immature lymphocytes, in whose development involves both environmental and genetic factors. It is well known that single nucleotide polymorphisms (SNPs) in coding and noncoding genes contribute to the susceptibility to ALL. This study aims to determine whether SNPs in *miR-146a*, *miR-196a-2*, *miR-499a*, and *miR-612* genes are associated with the risk to ALL in pediatric Mexican population.

**Methods:**

A multicenter case-control study was carried out including patients with *de novo* diagnosis of ALL and healthy subjects as control group. The DNA samples were obtained from saliva and peripheral blood, and the genotyping of rs2910164, rs12803915, rs11614913, and rs3746444 was performed using the 5′exonuclease technique. Gene-gene interaction was evaluated by the multifactor dimensionality reduction (MDR) software.

**Results:**

*miR-499a* rs3746444 showed significant differences among cases and controls. The rs3746444G allele was found as a risk factor to ALL (OR, 1.6 [95% CI, 1.05–2.5]; *p* = 0.028). The homozygous GG genotype of rs3746444 confers higher risk to ALL than the AA genotype (OR, 5.3 [95% CI, 1.23–23.4]; *p* = 0.01). Moreover, GG genotype highly increases the risk to ALL in male group (OR, 17.6 [95% CI, 1.04–298.9]; *p* = 0.00393). In addition, an association in a gender-dependent manner among SNPs located in *miR-146a* and *miR-196a-2* genes and ALL susceptibility was found.

**Conclusion:**

Our findings suggest that SNP located in *miR-499a*, *miR-146a*, and *miR-196a-2* genes confer risk to ALL in Mexican children. Experimental analysis to decipher the role of these SNPs in human hematopoiesis could improve our understanding of the molecular mechanism underlying the development of ALL.

## Introduction

Acute lymphoblastic leukemia (ALL) is the most common pediatric hematological malignancy around the world, representing over 80% of all cases under 18 years old (1). This entity is highly prevalent in Mexican population, which displays one of the highest rate of relapse and death in comparison with other ethnic groups even after using chemotherapeutic approaches implemented in developed countries (2, 3). ALL emerges by an abnormal proliferation of immature lymphocytes and their progenitors that replace the hematopoietic elements in the bone marrow and other lymphoid organs. So far, most of the causes of ALL are undeciphered; however, it is well known that an interaction within environmental and genetic factors is needed to develop this malignancy (4–6). Among the identified risk genetic factors to suffer ALL are the single nucleotide polymorphisms (SNP), both, in coding and no coding genes (6–9). No coding genes comprises around 98% of the human-transcribed genome, which is mainly represented by microRNAs (miRNAs) and long noncoding RNAs (lncRNAs) that play a relevant role in LLA and other types of cancer (10). miRNAs are small endogenous RNAs of 19–25 nucleotides that function as posttranscriptional regulators silencing specific mRNAs. miRNAs interact with their targeted mRNAs by complementary base pairing, most of them in the 3′-untranslated region (UTR) of the target mRNA, although interplay in the 5′UTR region has also been documented. Targeted coding mRNAs by specific miRNAs could be either in complete or incomplete fashion (11). Experimental evidences have revealed that miRNA dysfunction contributes to the establishment of diverse human diseases, since miRNA-mRNA-specific interaction makes fine-scale adjustments to protein outputs (8, 12, 13). It has been identified that several SNP located into miRNA gene sequences are closely responsive for their abnormal function by modifying pri-miRNA transcription, pri-miRNA/pre-miRNA processing, or by disrupting miRNA-mRNA interactions (14, 15). The rs2910164 G/C in *miR-146a* gene has been reported as an alterer of the gene expression, then its targeted mRNAs, which are involved in fundamental biological processes (cell differentiation, hematopoyesis, and innate and adaptive immunity, etc.) (16, 17). The rs2910164 has been associated with many types of cancer and several immune-mediated diseases (18–20); however, its association with ALL has shown controversial results (9, 17, 21). Another functional miR-SNP is rs3746444, which results from an A-to-G substitution in the seed region of *miR-499a*, was reported as significantly associated with an increased susceptibility to several human conditions, including cancer (19, 22). To know whether rs2910164 G/C in *miR-146a*, rs11614913 T/C in *miR-196a-2*, rs3746444 A/G in *miR-499a*, and rs12803915 G/A in *miR-612* are associated with ALL in Mexican children, we performed a case control study.

## Material and Methods

### Subjects

As part of the Mexican Interinstitutional Group for the Identification of the Causes of Childhood Leukemia (MIGICCL), we conducted a case-controls study from August 1, 2014, to July 31, 2016. Participants were younger than 18 years, residents of the Metropolitan Area of Mexico City, and recruited from public hospitals and health institutions from Mexico City, Mexico as was described previously by Medina-Sanzon et al. (6). ALL diagnosis was established by either a hematologist or an oncologist according to clinical characteristics, and bone marrow (BM) aspirate data. Gender, age at diagnosis, white blood cell count (WBC), immunophenotype, and risk classification group were registered from the patients’ medical records. We used the National Cancer Institute (NCI) risk criteria for ALL case stratification as follows: (a) standard risk: 1–9.99 years of age or WBC <50 × 10^9/L, and (b) high risk: ≤1 or ≥10 years of age and/or WBC ≥50 × 10^9/L. Patients included in the study were treated with chemotherapy, none of them received HSCT therapy. Relapse was considered when ≥5% leukemic blasts were detected in BM sample during the first 36 months after having achieved complete remission (CR). Early mortality was defined as the patient’s death during the first 24 months. Cases with Down syndrome were excluded from the analysis. All institutional committees of Ethics, Research, and Biosecurity of the participant institutions approved this study. Written informed consent was obtained from all participants and the children’s parents. Patients ≥8 years old gave their assent (when possible) to be included in the present study. Cases and controls were selected according to criteria described in a previous study (6). Briefly, controls were recruited from second-level hospitals of the same health institution that referred the children with ALL to the third-level care hospitals. Control children were recruited from the departments of ambulatory surgery, pediatrics, and ophthalmology; orthopedic outpatient clinics; and the emergency room of the referred hospitals and have no leukemia, hematological diseases, allergies, infections, and congenital malformations. A set of adult patients was included to test the associated SNP *miR-499a_* rs3746444. The group of adult patients and controls is described in the *Material and Methods* section in the **Supplementary Material**.

### DNA Extraction, SNP Selection, and Genotyping

Genomic DNA from saliva or peripheral blood was obtained according to the ORAGENE Purification Kit (DNA Genotek Inc., Kanata, ON, Canada) and the Gentra Kit (Gentra Systems Inc., Minneapolis, MN, USA) according to the manufacturer’s instructions. DNA purity and concentration were determined by sypectrofotometry (Nanodrop-1000). The rs2910164 (*miR-146a*), rs11614913 (*miR-196a-2*), rs3746444 (*miR-499a*), and rs12803915 (*miR-612*) were selected base on previous association studies in ALL and other malignancies (8, 9, 13, 17, 21, 23–26). Genotyping was performed using the 5′exonuclease technique and TaqMan MGB chemistry in a QuantStudio 5 system according to the manufacturer’s instructions (Thermo Fisher, Foster City, CA, USA). TaqMan probes used were C:15946974_10 (rs2910164), C:31185852_10 (rs11614913), C:_2142612_40 (rs3746444), and C:32062363_10 (rs12803915). PCR reaction contained 25 ng of genomic DNA, 2.5 µl of TaqMan master mix, 0.0625 µl of 40× assay mix, and ddH_2_O up to a final volume of 5 µl. The PCR protocol included denaturing at 95°C for 10 min, followed by 40 cycles of denaturing at 95°C for 15 s, and annealing and extension at 60°C for 1 min. Genotypes were assigned automatically by measuring the allele-specific fluorescence by using QuantStudio Design and Analysis software 5 for allelic discrimination (Applied Biosystems, Foster City, CA, USA). The overall genotype call rate was over 98.0% and 100% concordance of a subset of randomly repeated samples during the genotyping.

### Statistical Analyses

Hardy-Weinberg Equilibrium (HWE) test was performed using the FINETTI program (http://ihg.gsf.de/cgicbin/hw/hwa1.pl). Alleles and genotype frequencies were compared among groups by using Chi-square and Fisher’s exact tests (when appropriate) implemented in the STATCALC program (Epi Info v.6.02 software, Centers for Disease Control and Prevention, Atlanta, GA). By comparing cases and controls, all SNPs were evaluated under the codominant, dominant, and recessive genetic models using the FINETTI program. Bonferroni correction test was applied. The multifactor dimensionality reduction (MDR) software (V 3.0.2) was used to evaluate gene-gene interactions (27). All *p*-values ≤ 0.05 were considered statistically significant.

## Results

### Features of Studied Subjects

The present work included 678 subjects from Mexico City, of which, 423 were children with ALL, and 255 children non-ALL. The ALL children were followed up for at least 3 years (3–7) after initial diagnosis. Males were more frequent than females either in cases (57.9% *vs*. 42.1%, respectively) nor controls (54.7% v/s 45.2%, respectively), but differences were not statistically significant (*p* = 0.43). The proportion of children under 10 years old were higher in both groups, and a significant difference was detected among cases (62.2%) and controls (71.1%) (*p* = 0.02). Median age of ALL children was 9.09 (0–18) and 6.4 (0–17) of the control group. Overall, 68.3% had >90% blast in bone marrow; 91.2%, 6.9%, and 1.9% were pre-B, cell-T, and biphenotype, respectively. Available clinical data are shown in **Table 1**.

**Table 1:** Clinical characteristics of patients with acute lymphoblastic leukemia.

Features	Cases (*n* = 423)	
	*n*	%
**Gender**		
Male	245	57.9
Female	178	42.1
**Age group (years)**		
<1	9	2.1
1–9	258	61.0
≥10	156	36.9
**Age at diagnosis (years)**		
Median (min–max)	7.9 (0–18)	
**BM blast at diagnosis (%)**		
<90	135	31.7
≥90	288	68.3
Median (min–max)	85.3 (20–100)	
**Inmunophenotype**		
Pre-B Cell	386	91.2
Cell-T	29	6.9
Biphenotype	8	1.9
**NCI risk classification**		
Standard risk	214	50.6
High risk	209	49.4
**Relapse**		
No	346	81.8
Yes	77	18.2
**Relapse site**		
Isolated BM	52	67.5
Isolated CNS	17	22.1
BM and CNS	2	2.6
BM and CNS and eye	1	1.3
CNS and eyes	1	1.3
BM and testis	3	3.9
Ovary	1	1.3
**Death**		
No	364	86.0
Yes	59	14.0

WBC, whole blood cell count; BM, bone marrow; NCI, National Cancer Institute; CNS, central nervous system.

### Association Study

Except for *miR-146a*, the genotypes of *miR-196a-2*, *miR-499a*, and *miR-612* were in HWE in the control population. The association analysis between miRNA SNPs and ALL are described in **Table 2** and **Supplementary Table S1**. Case-control analysis including all children showed a significant association among *miR-499a* rs374644 with ALL (**Table 2**). *miR-499a* rs3746444G alelle observed an OR of 1.6 (95% CI, 1.008–2.5), *p* = 0.028. However, this significance did not remain after Bonferroni correction test. To note, under codominant model analysis AA *vs*. GG, statistical significance was found: OR, 5.3 (95% CI, 1.23–23.4); *p* = 0.01 (**Table 1**). Stratification analysis by gender observed that *miR-499a* rs3746444G is associated with ALL in a gender-dependent manner, being a risk factor to males (OR, 2.46 [95% CI, 1.31–4.60]; *p* = 0.0037) but no to girls (*p* = 0.95) (**Table 3**). Moreover, in comparison with AA genotype, GG genotype highly increases the risk to ALL (OR, 17.6 [95% CI, 1.04–298.9]; *p* = 0.00393) in males. Data are shown in **Table 3**.

**Table 2:** Association analysis among miR-499 rs3746444 and acute lymphoblastic leukemia.

	Children		OR [CI], *p*-value	Adults		OR [CI], *p*-value	All		OR [CI], *p*-value
	Control (%)	Cases *n* (%)		Control *n* (%)	Cases *n* (%)		Control *n* (%)	Cases *n* (%)	
***N***	255	416		180	71		435	489	
**Genotypes**									
AA	229 (89.8)	362 (87.0)		157 (87.2)	59 (83.1)		386 (88.7)	421 (86.1)	
AG	24 (9.4)	39 (9.3)		23 (12.8)	9 (12.7)		47 (10.8)	48 (9.8)	
GG	2 (0.8)	17 (4.8)		0 (0)	3 (4.2)		2 (0.5)	20 (4.1)	
**Alelles**			1.6 [1.05–2.5], 0.028^*^			1.7 [0.87–3.34], 0.11			1.58 [1.1–2.2], 0.01^*^
A	482 (94.5)	763 (91.4)		337 (93.6)	127 (89.4)		819 (94.1)	824 (91.0)	
G	28 (5.5)	73 (8.8)		23 (6.4)	15 (10.6)		51 (5.9)	88 (9.0)	
**Codominant**			5.3 [1.23–23.4], 0.01^*^			18.5 [0.94–364], 0.005			9.16 [2.1–39.4], 0.00033^*^
AA *vs*. GG									

OR, odds ratio; CI, confidence interval. ^*^Statistically significant.

**Table 3:** Association analysis among miR-499 rs3746444 and acute lymphoblastic leukemia in children stratified by gender.

	Male		OR [CI], *p*-value	Female		OR [CI], *p*-value
	Control (%)	Cases *n* (%)		Control *n* (%)	Cases *n* (%)	
***N***	255	416		180	71	
**Genotypes**						
AA	126 (89.8)	207 (87.0)		103 (87.2)	155 (83.1)	
AG	13 (9.4)	25 (9.3)		9 (12.8)	14 (12.7)	
GG	0 (0.8)	14 (4.8)		2 (0)	3 (4.2)	
**Alelles**			2.46 [1.31–4.60], 0.0037^*^			1.021 [0.49–2.09], 0.95
A	482 (94.5)	763 (91.4)		337 (93.6)	127 (89.4)	
G	28 (5.5)	73 (8.8)		23 (6.4)	15 (10.6)	
**Codominant**			17.6, [1.04–298.9], 0.00393^*^			0.99 [0.16 6.06], 0.99
AA *vs*. GG						

OR, odds ratio; CI, confidence interval. ^*^Statistically significant. Genotyping >98%.

*miR-146a* rs2910164, *miR-196a-2* rs11614913, and *miR-612* rs12803915 association analysis including all children with ALL showed differences among cases and controls but were not statistically significant (**Supplementary Table S1**). The analysis stratified by gender revealed that homozygote genotype for the minor allele CC of *miR-146a* rs2910164 was differentially distributed among male ALL cases and male controls (OR, 4.3 (1.60–11.61); *p* = 0.02). Meanwhile, *miR-196a-2* rs11619413 was associated with ALL in female (C *vs*. T: OR, 1.54 [95% CI, 1.08–2.2]; *p* = 0.015) (**Supplementary Table S2**).

### Association Between *miR-146a*, *miR-196a-2*, *miR-499a*, and *miR-612* SNPs With Clinical Characteristics

To know whether the studied SNPs were associated with clinical and biological ALL features, we performed the case-control analysis into the patients group stratified by gender, age group, immunophenotype, NCI-risk classification, relapse, death, and hereditary cancer family history (**Supplementary Table S3**). Significant differences among gender and age were found in the distribution of the *miR-196a-2* rs11614913C allele (*p* = 0.02, *p* = 0.02, respectively). Additionally, analysis comparing infants *versus* children older than 1 year was performed. **Supplementary Table S3** shows the results grouping the patients by age groups: <1 year; 1–9.9 and ≥10 years, considering that it has been reported that adolescents with ALL also have a dismal prognosis in comparison with children below this age and is considered an important prognostic factor. Regarding immunophenotype, NCI risk classification, relapse, death, and hereditary family history, no significant differences were observed (**Supplementary Table S3**). Furthermore, we conducted survival analyses between the SNPs analyzed and the overall survival of pediatric patients with ALL, but no significant associations were observed neither including all cases nor after stratifying by child’s sex and age groups.

### Gene-Gene Interaction Analysis

**Figure 1:**
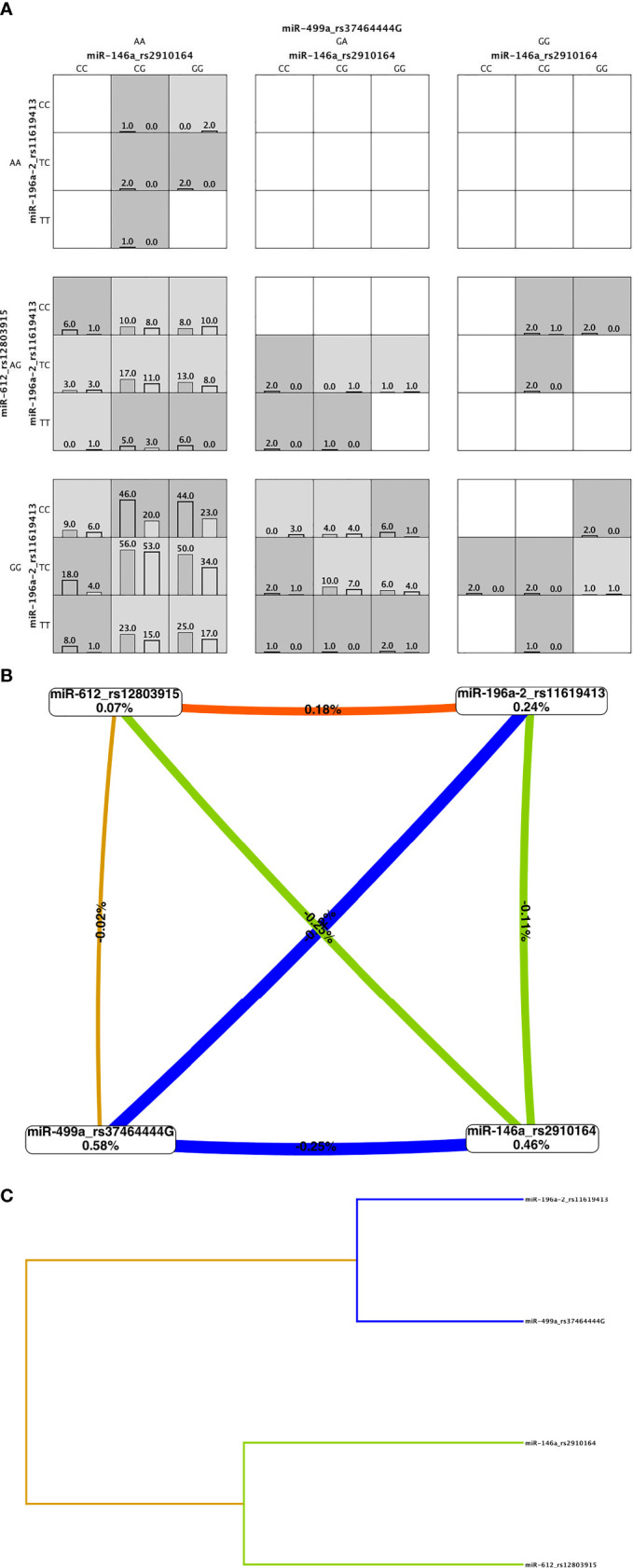
Multifactor dimensionality reduction (MDR) analysis. **(A)** Four-*locus* MDR model. Genotype combinations with high risk (shaded dark grey) and low risk (shaded light grey) for acute lymphoblastic leukemia (ALL) and their distribution in cases (left bar) and controls (right bar). The patterns of high (shaded and low-risk cells, which differ across each of the different multi locus dimension, means that the influence each genotype on the ALL risk is dependent on the genotypes a each of the other three loci. **(B)** Interaction entropy graph for gene-gene interaction and ALL risk. Graph shows the percent of the entropy in case-control removed by each factor (boxes) and by each pair-wise combination of attributes (lines). Positive value and orange line indicate low degree of synergy and negative values and blue and green lines mean redundancy. Gold line means independency. **(C)** The dendrogram graphic shows the presence, strength, and nature of epistatic effects. The shorter the line connecting two attributes the stronger the interaction. Strength of interaction goes from left to right (gray line).

To know whether **g**ene-gene interactions among *miR-146a*, *miR-196a-2*, *miR-499*a, and *miR-612* SNPs predict the risk to ALL, a MDR analysis was performed by including cases and controls having complete genotyping data of all evaluated SNPs. No SNP was identified as the best factor model. The multi*locus* model with maximum crossvalidation consistency (CVC) and minimum prediction error is displayed in **Supplementary Table S4**. Four-*locus* genotype combinations associated with the risk of ALL, as well as their distribution among cases (left) and controls (right) is summarizes in **Figure 1A**. This analysis gave evidence of epistasis or gene-gene interaction (**Figures 1B, C**). Entropy data showed that rs3746444 had the larger effect on the susceptibility to develop ALL (0.59%) followed by rs*2910164* (0.49%). Week synergy among *miR-196a-2* and *miR-612* was observed (orange line) (**Figure 1B**). Redundancy was observed among all SNPs (blue and green lines) (**Figures 1B, C**). To note, gene-gene gender interaction observed a strong synergy (red line) among *miR-196a-2* and gender (**Supplementary Figure S1**).

## Discussion

Mountain evidence reveals that miRNAs are relevant in the gene regulation contributing to the establishment of human diseases and modifying their treatment response of the patients. For instance, by using miRanda, TargetScan, and miRTarget2, it is predicted that *AKT2* is a potential target of *miR-612*, which has been reported as significantly upregulated in ALL patients. *AKT2* expression in lymphocytes correlates negatively with sensitive to glucocorticoids, and patients have poor prognosis (28–30). For its part, *miR-146a* has been involved in megakaryopoiesis by activating innate immunity targets TIRAP and TRAF6 (31). In addition, experimental data have shown that SNPs in miRNAs could affect cell differentiation, proliferation, and apoptosis conducting cancer development. The SNPs rs2910164 in *miR146a*, rs11614913 in *miR-196a-2*, rs3746444 in *miR-499*a, and rs12803915 in *miR-612* are among the most studied SNPs in cancer. In a case-control study, we did no find association among rs12803915 of *miR-612* but to rs3746444 of *miR-499a* with ALL, as well as, in a gender-dependent manner rs2910164 of *miR146a*, and rs11614913 of *miR-196a-2* were associated with the risk to this disease.

To date, only three studies have explored the association among *miR-499a* rs3746444 and ALL. Our results are in line with the findings of de Souza et al., who studied 100 pediatric ALL patients, and 180 healthy individuals from Brazilian-amazon reported that *miR499a*_rs3746444 increases 17-fold the risk of development of ALL (26). We found that the mutant homozygote rs3746444GG genotype was associated with a 1.6-fold increase in the risk of developing ALL. However, our data are in contrast to those published previously by Gutierrez-Camino et al., who including 213 B-cell ALL pediatric patients and 387 controls from Spain, found a protective role of the G allele on the risk of ALL (8) and by Hasani et al., studying 75 children diagnosed with ALL and 115 children from Iran with no history of any type of cancer (23). To note, we explored whether *miR-499a* rs3746444 has in adults with ALL the same effect as we observed in children by genotyping 71 patients >18 years old with clinical diagnosis of ALL and 180 healthy adults (1:1 female/male). Samples from ALL adults were obtained from the biobank of the Servicio de Hematología, Hospital General de México. Adult control group was obtained from the DNA biobank of the laboratorio de Investigation, Hospital Juárez de México. *miR-499a* rs3746444A allele frequency was very similar among children and adults (cases and controls) and notably, *miR-499a* rs3746444G allele was not detected in no-ALL adults (0%). However, differences among adult cases and adult controls or between children and adults were not statistically significant (**Table 2**). Our study is the first to investigate the role of rs3746444 in the susceptibility to ALL in adults, which has been associated with common adulthood cancer types (22, 32). The rs3746444 is located in pre-*mir-499* gene resulting changes of an A:U to a G:U pairing and mismatching that reduces the stability of the *pre-miR-499* secondary structure (33) and this SNP, located in the seed region of miR-499a could alter the targeted genes. In fact, Yang et al. (34) reported that this SNP potentially recognizes 573 new target genes and lost 5,392 original target genes. Several of these genes are involved in biological processes as cell proliferation and migration (35).

It is known that *mir146a* plays anti-inflammatory functions, has roles as tumor suppressor and commonly shows altered expression levels in human leukemia (32–38). Data from ALL Jurkat cells have shown that *miR-146*a can promote growth of leukemia cells by regulating the expression anti-apoptosis factor Bcl-xL and altering the expression of diverse genes involved in T-cell differentiation (37–39). Recent papers have given evidence that rs2910164 in *miR146* can modify the expression of nuclear factor (NF-ĸB) through reducing *IRAK1* and *TRAF* gene expression thus, driving inflammation and leukemia progression in myeloid cells (40). Stickel et al. (41) observed that patients with the *miR-146a* polymorphism rs2910164 display higher major histocompatibility complex class II (MHCII) molecule levels on monocytes. In addition, experimental evidences have shown that the rs2910164 in human allogeneic hematopoietic cell transplantation (allo-HCT) recipients significantly increases the risk for acute severe acute graft-*versus*-host disease in patients with hematological malignancies (41). The G to C polymorphism rs2910164 in *miR146a* changes the G:U pair to a C:U mismatch in the stem structure of *miR-146a* precursor, resulting in a reduced level of mature miR146a (36). To note, we found that *miR-146a* rs2910164 GG genotype confer risk to ALL in male. This SNP is widely associated with cancer, but association studies in ALL have reveled conflicting results. On one hand, it has been reported that *miR-146a* rs2910164 is associated with childhood ALL susceptibility in Asian population, including Iranian, Chinese, and Taiwanese (17, 23, 25). On the other hand, studies in Thailand, India, and China failed to replicate these results (9, 21, 42). No published study has reported an association among ALL and rs2910164 in a gene-dependent manner, and considering the higher prevalence of ALL in male than female, these findings should be deeply explored.

Regarding rs11614913 C/T, in the 3p mature miRNA region of *miR-196a2*, leads to a variation from G:T to G:C in the stem region of the *miR-196a2* precursor. Comparing the minimum free energy for optimal secondary structures of the SNP rs11614913 in pre-miR196a2 found that this SNP had no dramatic effect on its secondary structure (43); however, Hoffman et al. (44) already show that rs11614913C may affect the processing of pre-miRNA, modify both, its expression level and function, then alters its interactions with its targeted genes. In fact, various studies have observed a correlation among abnormal expression of *miR-196a2* and genes involved in cancer (45, 46). Studies in several types of cancer suggest that the common rs11614913 variant may play a role in the development of malignancies in an ethnic-dependent manner (43, 47, 48). For instance, a meta-analysis including 41,673 cases and 49,570 controls from 111 studies revealed that *mir-196a-2* rs11614913 T allele was significantly associated with cancer risk only in Asians but not Caucasians (47). As for hematological malignancies, association data are scarce. Findings in Non-Hodgkin’s lymphoma suggest that the *miR-196a-2* polymorphism may increase the risk of the disease by altering the expression of mature *miR-196a* (48). In ALL, two studies have published that rs11614913C allele contributes to an increased risk of this disease in Thailand, and China, but another one found no association results in Taiwanese ALL cases (13, 24, 49). Comparing the minimum free energy for optimal secondary structures of the SNP rs11614913 in *pre-miR196a-2* found no dramatic effect on its secondary structure (47). We found an association among this SNP with ALL risk in females, but whether this SNP is playing a role in ALL susceptibility remains unknown.

Regarding rs12803915 in *mir-612*, experimental studies reveal that rs12803915 SNP affects mature *mir-612* expression in a cell-type-specific manner. As example, Kim et al. observed that rs12803915A allele increases and decreases mature *mir-612* expression in prostate cancer and colon cancer cell lines, respectively (50). In ALL, two studies have explored this SNP (8, 51). On one hand, the rs12803915 in *mir-612* was associated with ALL in patients from Spain (8). On the other hand, in 100 B-ALL cases and 105 controls from Iran, no association was observed (51).

To know whether there is a gene-gene interaction among the evaluated SNPs in the risk to ALL, we employed a MDR analysis. We observed that *miR-499a* is the main casual factor for ALL, a strong redundancy interaction effect of this SNP and *miR-196a-2* and *miR-146a* on ALL risk, and a low synergism with *miR-612*; thus, this analysis gave evidence of epistasis. Both genes have already been shown to be associated with cancer risk in various populations, but no data regarding their interaction has been published. To note, both SNPs have been found as susceptibility factors to ALL in a Spanish population (8).

The discrepancies on the association findings among the present work and other populations may be related to the sample selection, and the genetic background of the populations, since the linkage disequilibrium complex structure of the populations could mask the causal SNP (51). In addition, differences in the genetic background of cases and control could bias the association results. To note, our control group and a subset of the ALL cases belong to a genotyped cohort using 32 informative ancestry markers. As we published previously, ALL cases and controls are Mexican-Mestizo (6). However, to clarify the effect of miRNA polymorphism on ALL risk, studies including patients from different ethnicities and larger sample sizes are needed. Experimental analysis could also add data to decipher the role of *miR-499* in ALL.

In conclusion, our analysis revealed that *miR-499* rs3746444 confers risk to ALL and there is a gender-dependent association among *miR-146a* and *miR-196a-2* and ALL in Mexican children. Studies are needed to evaluate the potential molecular mechanisms underlying the contribution of these SNPs in ALL susceptibility.

## Data Availability Statement

The original contributions presented in the study are included in the article/**Supplementary Material**. Further inquiries can be directed to the corresponding authors.

## Ethics Statement

The studies involving human participants were reviewed and approved by the Ethics and National Committee of Scientific Research of the Instituto Mexicano del Seguro Social with number R-2013-785-062. Written informed consent to participate in this study was provided by the participants’ legal guardian/next of kin.

## Author Contributions

SJ-M: conceptualization. SJ-M, JN-E, JC-I, and JR-B: methodology. SJ-M, JN-E, JC-I, and JR-B: formal analysis. SJ-M: investigation. JN-E, VB-M, EJ-H, AM-S, IO-C, AM-T, JF-L, MP-S, JM-T, HP-L, RA-S, FM-R, JP-G, DD-R, JT-N, JF-B, RE-E, PR-Z, LF-V, ET-G, VL-G, JL-R, JG-U, SM-S, GE-A, CA-H, RR-C, LH-M, LG-L, GC-O, AG-E, IC-H, AM-H, ML-C, NH-P, JG-K, MR-V, DT-V, CC-R, FM-L, JP-G, AM-R, AA-S, BS-D, MG-R, LM-P, GV-A, MM-R, OS-R, HR-V, JR-B, and AH-M: resources. SJ-M: writing—original draft preparation. SJ-M, AH-M, and JM-A: writing—review and editing and supervision. SJ-M, JC-E, and JM-A: funding acquisition. All authors reviewed the final manuscript and read and approved the submitted version.

## Funding

This work was supported by the Consejo Nacional de Ciencia y Tecnología (CONACyT), Investigación en Fronteras de la Ciencia (IFC)-2016-01-2119, PDCPN2013-01-215726, CB-2015-1-258042, FIS/IMSS/PROT/1548, FONCICYT/37/2018, FIS/IMSS/PROT/1782, and FORDECYT-PRONACES-377883-2020. We also thank the financial support from the National Institute of Genomic Medicine (01/2018/I, 19/2019/I).

## Conflict of Interest

The authors declare that the research was conducted in the absence of any commercial or financial relationships that could be construed as a potential conflict of interest.

## Publisher’s Note

All claims expressed in this article are solely those of the authors and do not necessarily represent those of their affiliated organizations, or those of the publisher, the editors and the reviewers. Any product that may be evaluated in this article, or claim that may be made by its manufacturer, is not guaranteed or endorsed by the publisher.
