# Supplementary material for: Association Analysis Between the Functional Single Nucleotide Variants in miR-146a, miR-196a-2, miR-499a, and miR-612 With Acute Lymphoblastic Leukemia
Source: Front Oncol. 2021 Nov 5;11:762063. doi: 10.3389/fonc.2021.762063 (PMC8602911; doi:10.3389/fonc.2021.762063)
Supplement: Supplementary file 1 [file DataSheet_1.docx]

Supplementary Material

**Supplementary Material and Methods**

**Adults, population studied.**

DNA samples from adults with clinical diagnosis of ALL were obtained from the biobank of the Servicio de Hematología, Hospital General de México. These samples were collected from bone marrow of *de novo* ALL. Adult cases were 48.6% female and 51.4% male and mean age 35.03 (SD +15) years old, and all patients were treated with chemotherapy. DNA sample of adult with no-ALL were obtained from the DNA biobank of the Laboratorio de Investigacion, Hospital Juárez de México. These DNA samples were extracted from blood unrelated donors, over 18 years of age, with self-reported Mexican-Mestizo ancestry (three generations), and with no family history of autoimmune or chronic inflammatory diseases. Controls were 50%:50% female/male.

**Supplementary tables**

**Supplementary table 1**. Association analysis among SNPs in miR-146, miR-196A2, and miR-612 genes in children with acute lymphoblastic leukemia

| **Gen** | **ID SNP** | **Genotypes / alleles** | **Children** | | **OR [95% CI],** P value |
| --- | --- | --- | --- | --- | --- |
|  |  |  | **Control**n (%) | **Cases**N (%) |  |
| **miR-146** | rs2910164 | GGGCCCGC | 105 (41.2)130 (51.0)20 (7.8)340 (66.7)170 (33.3) | 175 (41.7)191 (45)54 (13)541 (64.4)299 (35.6.) | 1.1 [0.87-1.39], 0.49 |
| **miR-196A2** | rs11614913 | CCCTTTCT | 81 (32.1)132 (52.4)39 (15.5)294 (58.3)210 (41.7) | 141 (33.9)195 (46.9)80 (19.2)477 (57.3)355 (42.7) | 1,04 [0.83-1.3], 0.71 |
| miR-612 | rs12803915 | GGGAAAAG | 200 (79.4)50 (19.8)2 (0.8450 (89.3)54 (10.7) | 329 (79.1)81 (19.5)6 (1.4)739 (88.8)93 (11.2) | 1.04 [0.73-1.49], 0.79 |

SNPs: Single nucleotide polymorphism; OR: odds ratio; CI: confidence interval; P: p value, >98% of genotyping in all SNPs.

**Supplementary table 2**. Case-control analysis among SNPs in *miR-146*, *miR-196a-2*, and *miR-612* genes by gender in children with acute lymphoblastic leukemia.

| **Gen** | **ID SNP** | **Genotypes / alleles** | **Male** | | **OR [95% CI],**  **P value** | **Female** | | **OR [CI],**  **P value** |
| --- | --- | --- | --- | --- | --- | --- | --- | --- |
|  |  |  | **Control**  **n (%)** | **Cases**  **N (%)** |  | **Control**  **n (%)** | **Cases**  **N (%)** |  |
| ***miR-146a*** | rs2910164* | GG  GC  CC  G  C  GG vs CC | 60 (41.2)  76 (51.0)  5 (7.8)  340 (66.7)  170 (33.3) | 100 (41.7)  110 (45)  36 (13)  541 (64.4)  299 (35.6.) | 1.3[0.97-1.82], 0.06  4.3 [1.60-11.61], 0.002* | 45 (41.2)  54 (51.0)  15 (7.8)  340 (66.7)  170 (33.3) | 75 (41.7)  81 (45)  18 (13)  541 (64.4)  299 (35.6.) | 0.86 [0.61-1.23], 0.42  0.72 [0.33-1.56], 0.40 |
| ***miR-196a-2*** | rs11614913* | CC  CT  TT  C  T  CC vs TC  CC vs TT  CC vs [CT + TT | 40 (32.1)  79 (52.4)  25 (15.5)  294 (58.3)  210 (41.7) | 94 (33.9)  108 (46.9)  42 (19.2)  477 (57.3)  355 (42.7) | 0.79 [0.59-1.07], 0.13  0.58 [0.36-0.93], 0.023*  0.71 [0.38-1.32], 0.28  0.61 [0.39-0.95], 0.03* | 41 (32.1)  47 (52.4)  14 (15.5)  294 (58.3)  210 (41.7) | 47 (33.9)  87 (46.9)  38 (19.2)  477 (57.3)  355 (42.7) | 1.54 [1.08-2.2], 0.015*  1.61 [0.93-2.79], 0.08  2.36 [1.12-4.97], 0.021*  1.78 [1.06-3.00], 0.027* |
| ***miR-612*** | rs12803915* | GG  GA  AA  A  G | 116 (79.4)  24 (19.8)  0 (0.8  450 (89.3)  54 (10.7) | 189 (79.1)  49 (19.5)  6 (1.4)  739 (88.8)  93 (11.2) | 1.52 [0.97-2.50], 0.09 | 84 (79.4)  26 (19.8)  2 (0.8  450 (89.3)  54 (10.7) | 140 (79.1)  32 (19.5)  0 (1.4)  739 (88.8)  93 (11.2) | 0.66 [0.39-1.12], 0.12 |

OR: odds ratio; CI: confidence interval; *: statistically significant. ºGenotyping >98%.

**Supplementary table 3**. Association analysis stratifying by clinical and demographic features in children with ALL.

| **Features** |  | **miR-146a rs2910164º** | | | **P** | **miR-196a-2 rs11614913º** | | | **P** | **miR-499 rs3746444º** | | | **P** | **miR-612 12803915º** | | | **P** |
| --- | --- | --- | --- | --- | --- | --- | --- | --- | --- | --- | --- | --- | --- | --- | --- | --- | --- |
|  |  | GG | GC | CC |  | CC | CT | TT |  | AA | AG | GG |  | GG | GA | AA |  |
| Gender | Male | 100 (40.7) | 110 (44.7) | 36 (14.6) | 0.31 | 94 (38.5) | 108 (44.4) | 42 (17.2) | 0.02* | 207 (84.1) | 25 (10.2) | 14 (5.7) | 0.01* | 189 (77.4) | 49 (20.1) | 6 (2.5) | 0.14 |
|  | Female | 75 (43.1) | 81 (46.6) | 18 (10.3) |  | 47 (27.3) | 87 (50.6) | 38 (22.1) |  | 155  (90.1) | 14  (8.1) | 3  (1.8) |  | 140 (81.4) | 32 (18.6) | 0  (0) |  |
| Age at diagnosis | <1 | 4 (41.6) | 4 (51.9) | 1  (6.5) | 0.93 | 3 (28.0) | 4 (57.4) | 2 (17.6) | 0.17 | 9 (92.4) | 0  (6.5) | 0  (1.1) | 1.0 | 6 (79.7) | 3 (17.8) | 0 (0.5) | 0.50 |
|  | 1-9.9 | 77 (41.6) | 96 (51.9) | 12  (6.5) | Ref. | 51 (28.0) | 99 (57.4) | 32 (17.6) | Ref. | 171 (92.4) | 12  (6.5) | 2  (1.1) | Ref. | 145 (79.7) | 36 (17.8) | 1 (0.5) | Ref. |
|  | >10 | 28 (39.4) | 35 (49.3) | 8  (11.3) | 0.45 | 30 (42.9) | 33 (47.1) | 7 (10.0) | 0.02* | 58 (82.9) | 12 (17.1) | 0  (0) | 0.06 | 55 (75.6) | 14 (20.0) | 1 (1.4) | 0.74 |
| HCFH | No | 123 (42.9) | 131 (45.6) | 33 (11.5) | 0.25 | 94 (33.1) | 133 (46.8) | 57 (20.1) | 0.48 | 253 (88.2) | 24  (8.3) | 10 (3.5) | 0.1 | 216 (76.3) | 63 (22.3) | 4 (1.4) | 0.06 |
|  | Yes | 52 (39.1) | 60 (45.1) | 21 (15.8) |  | 47 (35.6) | 62 (47.0) | 23 (17.4) |  | 109 (83.2) | 15 (11.5) | 7  (5.3) |  | 113 (85.0) | 18 (13.5) | 2 (1.5) |  |
| Immunophenotype | B-cellprecursor | 158 (41.3) | 179 (46.7) | 46 (12.0) | Ref. | 124 (32.6) | 182 (47.9) | 74 (19.5) | Ref. | 331 (86.9) | 36 (9.5) | 14 (3.6) |  | 301 (79.4) | 72 (19.0) | 6 (1.6) | Ref. |
|  | T-cell | 14 (48.3) | 9 (31.0) | 6 (20.7) | 0.89 | 15 (55.6) | 6 (22.2) | 6 (22.2) | 0.14 | 25 (86.2) | 2(6.9) | 2(6.9) | 0.62 | 21 (72.4) | 8 (27.6) | 0 (0.0) | 0.52 |
|  | Biphenotypic | 3 (37.5) | 3 (37.5) | 2 (25) | 0.48 | 2 (25) | 5 (62.5) | 1 (12.5) | 0.63 | 6 (75.0) | 1 (12.5) | 1 (12.5) | 0.30 | 8 (100) | 0 (0.0) | 0 (0.0) | 0.36 |
| Risk | Standard | 82 (38.5) | 99 (46.5) | 32 (15.0) | 0.1 | 64 (30.2) | 107 (50.5) | 41 (19.3) | 0.25 | 185 (86.9) | 17  (8.0) | 11 (5.1) | 0.65 | 162 (76.5) | 47 (22.3) | 3 (1.2) | 0.21 |
|  | High | 93 (44.9) | 92 (44.5) | 22 (10.6) |  | 77 (37.7) | 88 (43.2) | 39 (19.1) |  | 177 (86.3) | 22 (10.7) | 6  (3.0) |  | 167 (81.9) | 34 (16.7) | 3 (1.4) |  |
| Relapse | No | 144 (41.9) | 156 (45.3) | 44 (12.8) | 0.86 | 117 (32.2) | 159 (46.5) | 66 (19.3) | 0.87 | 298 (86.9) | 33  (9.6) | 12 (3.5) | 0.35 | 267 (78.5) | 68 (20.0) | 5 (1.5) | 0.57 |
|  | Yes | 31 (40.8) | 35 (46.1) | 10 (13.1) |  | 24 (32.4) | 36 (48.6) | 14 (19.0) |  | 64 (85.3) | 6  (8.0) | 5  (6.7) |  | 62 (81.6) | 13 (17.1) | 1 (1.3) |  |
| Death | No | 155 (42.8) | 160 (44.2) | 47 (13.0) | 0.43 | 120 (33.5) | 167 (46.5) | 72 (39.0) | 0.34 | 310 (86.4) | 34  (9.5) | 15 (4.1) | 0.64 | 286 (79.4) | 69 (19.2) | 5 (1.4) | 0.63 |
|  | Yes | 20 (34.5) | 31 (53.4) | 7  (12.1) |  | 21 (36.9) | 28 (49.1) | 8 (14.0) |  | 52 (88.1) | 5  (8.5) | 2  (3.4) |  | 43 (7.1) | 12 (2.1) | 1 (1.8) |  |

HCFH: hereditary cancer family history, ºGenotyping: >98%, *: statistically significant.

**Supplementary table 4**. Gene-Gene interaction analysis between *miR-146a*, *miR-196a-2*, *miR-499a*, and *miR-612* and acute lymphoblastic leukemia risk prediction.

| **Model** | **TA** | **TBA** | **CVC** |  | **Pª value** |
| --- | --- | --- | --- | --- | --- |
| miR-146a_rs2910164 | 0.531 | 0.4762 | 6/10 | 1.22 (0.89-1.68) | < 0.2057 |
| miR-146a_rs2910164,miR-196a-2_rs11614913 | 0.5497 | 0.478 | 9/10 | 1.44 (1.04-1.99) | 0.024 |
| miR-146a_rs2910164,miR-196a-2_rs11614913,miR-612_rs12803915 | 0.5803 | 0.5221 | 10/10 | 1.99 (1.40-2.85) | < 0.0001 |
| miR-146a_rs2910164,miR-196a-2_rs11614913,miR-499a_rs3746444,miR-612_rs12803915 | 0.5999 | 0.5131 | 10/10 | 2.44 (1.70-3.48) | < 0.0001 |

Best one-to-four locus model *miR-146a*, *miR-196a-2*, *miR-499a*, and *miR-612* gene-gene interaction with cross-validation consistency (CVC) and prediction error per n-locus model obtained by multi factor dimensionality reduction (MDR) of our data set. TA: training balance accuracy; TBA: testing balance accuracy. ªValues from Whole Statistics data set.

**Supplementary Figures**

1. **
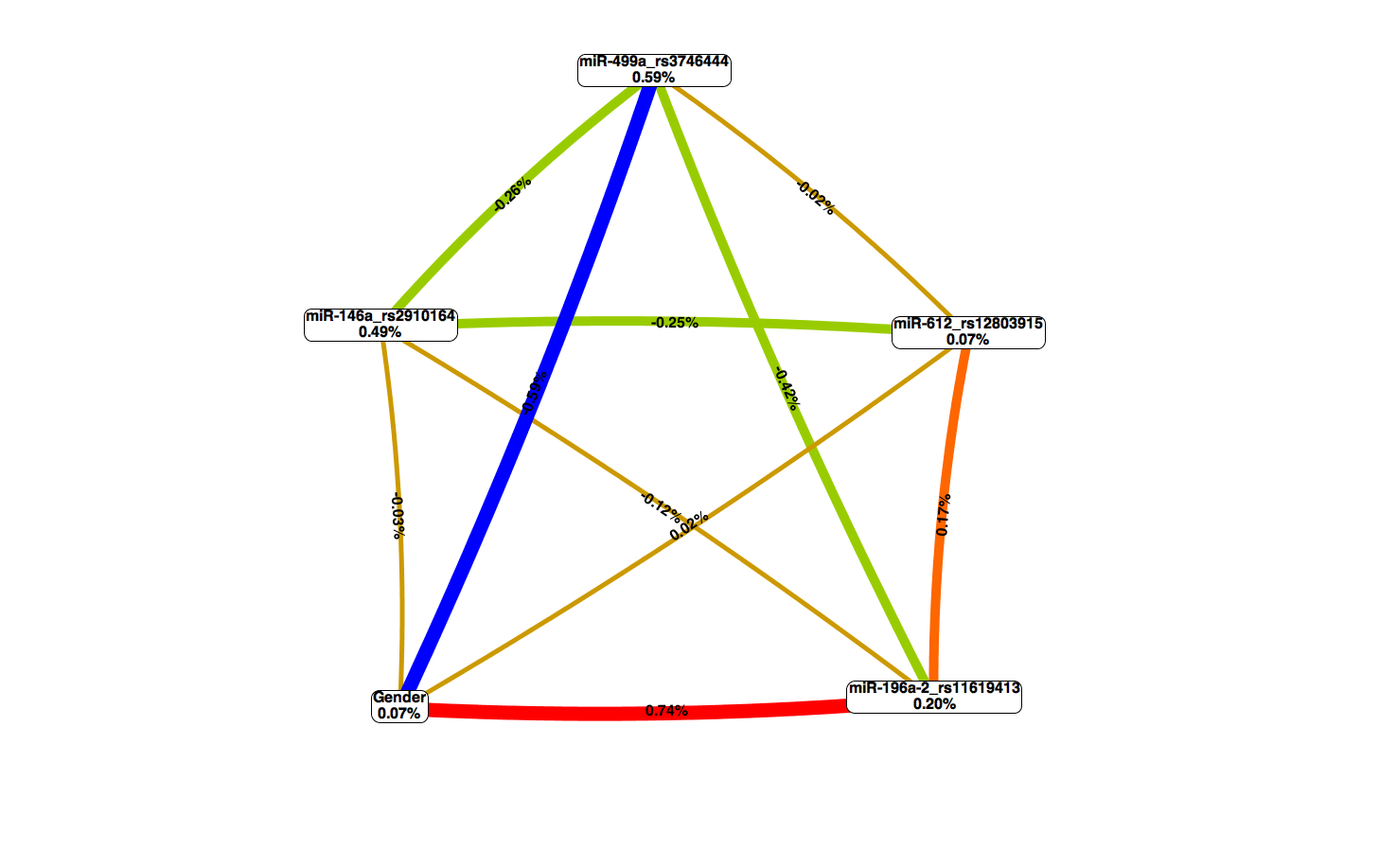
**

B)


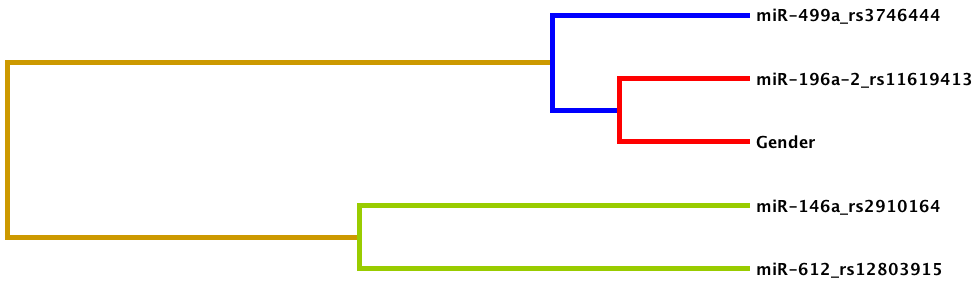


**Supplementary Figure 1.** Multifactor dimensionality reduction (MDR) analysis. A) Interaction entropy graph for gene-gene-gender interaction and ALL risk. Graph shows the percent of the entropy in case-control removed by each factor (boxes) and by each pair-wise combination of attributes (lines). Positive value indicate synergy and negative values mean redundancy. Graph shows a synergy effect among *miR-196a-2* rs11619413 and gender in the risk to ALL. B) The dendrogram graphic shows the presence, strength, and nature of epistatic effects. The shorter the line connecting two attributes the stronger the interaction. Strength of interaction goes from left to right (gray line). Red lines: strong synergy, orange line: low synergy, gold line: independency, blue and green lines: redundancy.
